# PSAFM: A parameter-free spatial attention fusion module

**DOI:** 10.1371/journal.pone.0357958

**Published:** 2026-09-15

**Authors:** Guangjun Liu, Xiaoping Xu, Bingkun Zhou, Feng Wang, Yike Li

**Affiliations:** 1 School of Ecological Environment and Chemical Engineering, Xi’an University of Technology, Xi’an, P. R. China; 2 School of Mathematics, Xi’an University of Technology, Xi’an, P. R. China; 3 School of Mathematics and Statistics, Xi’an Jiaotong University, Xi’an, P. R. China; 4 Department of Thyroid and Breast Surgery, Xijing Hospital of Air Force Military Medical University, Xi’an, P. R. China; Shijiazhuang Tiedao University, CHINA

## Abstract

Attention mechanisms improve convolutional neural networks (CNNs) by emphasizing informative features, but many existing modules introduce additional parameters, convolutional or fully connected layers, and non-negligible computational overhead. These costs may limit their use in lightweight or plug-and-play CNN architectures. To address this issue, this paper proposes a parameter-free spatial attention fusion module (PSAFM). Specifically, the pointwise average- and max-pooling branches are combined by deterministic weighted fusion, after which channel statistics and a Tanh activation are used to compute adaptive three-dimensional attention weights. This enables the module to strengthen feature responses without adding trainable parameters. We evaluate PSAFM on CIFAR-10 and CIFAR-100 using ResNet, Pre-ResNet, and MobileNetV2 backbones. On CIFAR-10, PSAFM achieves the best accuracy in 8 of 9 tested backbone settings and improves the original backbones by 0.32 to 1.81 percentage points. On CIFAR-100, PSAFM achieves the best accuracy in 6 of 9 settings and improves the original backbones by 0.05 to 1.68 percentage points. In these experiments, PSAFM is compared with representative attention modules. The results show that its parameter count remains essentially unchanged and that only a small FLOPs overhead is introduced. In other words, when model compactness is important, PSAFM is a simple and efficient attention module that improves CNN feature representation.

## Introduction

Convolutional Neural Networks (CNNs) remain a central architecture family in computer vision because they can learn hierarchical representations for classification, detection, segmentation, and related visual tasks. Since the success of AlexNet in large-scale image recognition [1], deeper and wider CNNs such as VGG [2] have substantially improved recognition accuracy. However, simply increasing network depth or width can also aggravate training difficulties, including vanishing gradients and network degradation [3,4]. Architectures such as ResNet, DenseNet, MobileNet, and EfficientNet have therefore been developed to improve optimization, feature reuse, and computational efficiency [3,5–7].

Beyond backbone design, attention mechanisms have become an important way to improve CNN representation by helping the model emphasize informative channels, spatial regions, or long-range relationships. The squeeze-and-excitation network (SE) recalibrates channel responses [8], the convolutional block attention module (CBAM) sequentially combines channel and spatial attention [9], and coordinate attention (CA) introduces coordinate-aware channel-spatial encoding for efficient mobile networks [10]. The simple parameter-free attention module (SimAM) further shows that useful attention weights can be derived without additional trainable parameters [11]. Recent work also continues to investigate efficient attention and feature reconstruction strategies for vision models [12–15].

Existing attention modules follow different feature-selection strategies. Channel-only modules emphasize inter-channel responses, spatial modules focus on localized regions, and channel-spatial modules combine both dimensions through additional branches or transformations. Parameter-free modules instead derive attention directly from feature statistics. These complementary design choices motivate a compact module that jointly exploits spatial and channel information while preserving the original parameter count. Table 1 summarizes the key characteristics of representative attention modules.

**Table 1:** Comparison of representative attention modules.

Module	Main operators	Attention type	Extra params	Key characteristic
SE	AP, FC, ReLU	Channel	2*C*^2^/*r*	Channel recalibration
CBAM	AP, MP, FC, Conv, BN	Channel-spatial	$2C^{2}/r+2k^{2}$	Sequential channel-spatial attention
CA	Directional AP, Conv, BN, activation	Coordinate channel-spatial	2*C*	Coordinate-aware feature encoding
SimAM	Energy function, ⊙	3-D attention	0	Parameter-free energy weighting
EMA	Grouped AP, Conv, GN, Sigmoid	Multi-scale channel-spatial	10(*C*/*g*)2 + 4*C*/*g*	Cross-spatial multi-scale aggregation
ELA	Strip AP, Conv, GN, Sigmoid	Directional spatial	(*k* + 2)*C*	Position-aware local attention
PSAFM	${\text{AP}}_{1\times 1}$/${\text{MP}}_{1\times 1}$, fusion, Tanh	3-D reweighting	0	Parameter-free fusion and 3-D reweighting

To address these challenges, this paper proposes a parameter-free spatial attention fusion module (PSAFM). The proposed PSAFM applies deterministic weighted fusion to pointwise average- and max-pooling branches and then derives adaptive attention weights from a simple channel-statistical energy formulation. This design avoids fully connected layers and convolutional attention branches; therefore, when PSAFM is inserted into an existing residual or inverted residual block, the parameter count remains essentially unchanged, which meets the design objective of this work.

**Fig 1:**
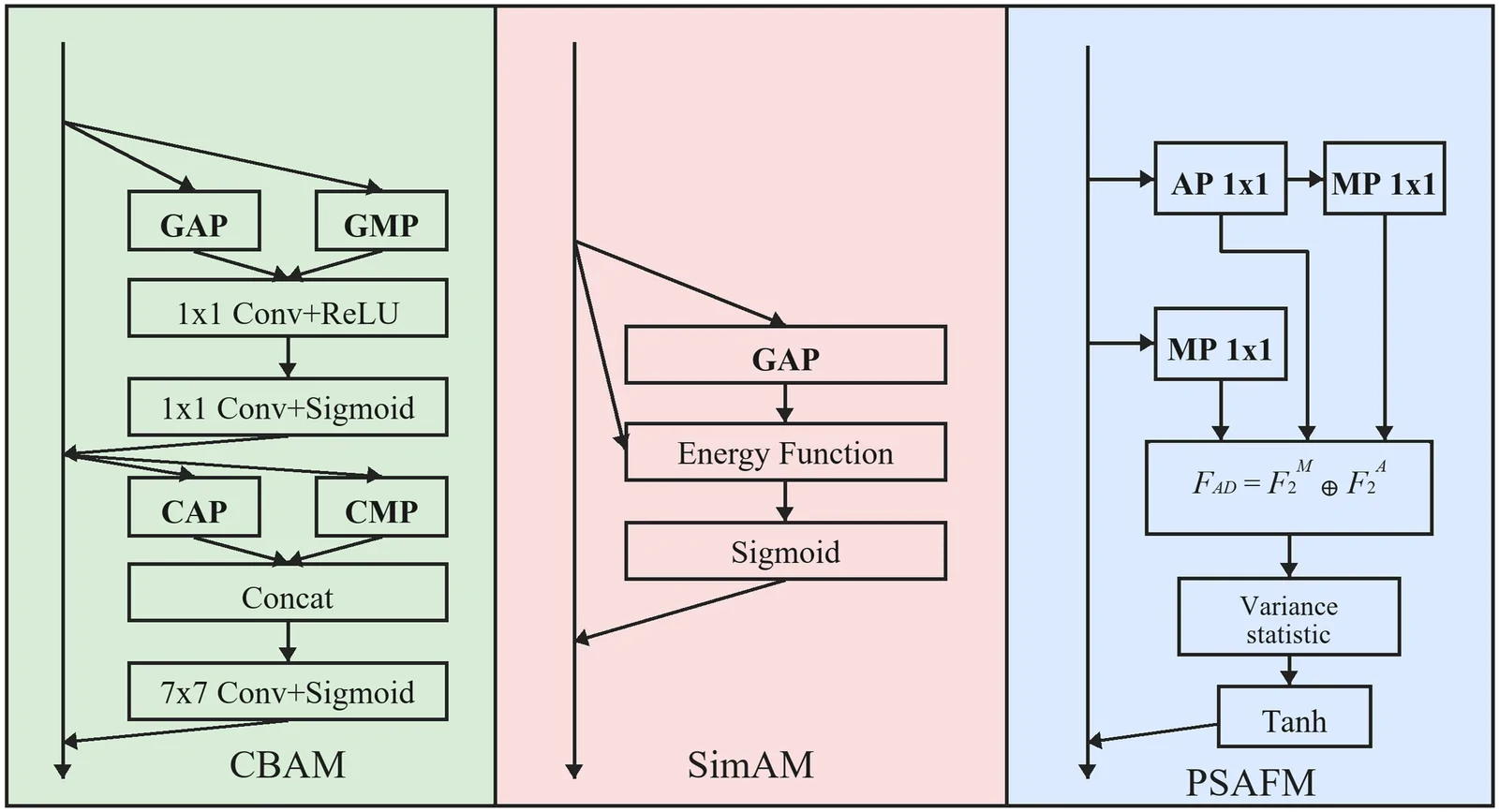
Design diagrams of different attention models. GAP and GMP represent global average pooling and global max pooling, respectively. CAP and CMP represent channel-wise average pooling and channel-wise max pooling, respectively. ${\text{AP}}_{1\times 1}$ and ${\text{MP}}_{1\times 1}$ denote the pointwise average- and max-pooling operators used in PSAFM. In the PSAFM panel, the pooling branches are fused before the variance-statistic and Tanh reweighting steps. Conv denotes a convolutional module, and Tanh denotes the activation used by PSAFM.

We emphasize that our work centers on creating a compact, plug-and-play attention module rather than proposing an entirely new architecture that departs from conventional CNN frameworks. In contrast to previous efforts that sought to integrate complex structures for attention inference, our approach provides a flexible, modular solution that retains a lightweight profile. Overall, the proposed attention mechanism combines spatial and channel information, and its practicality and effectiveness are demonstrated through experiments on multiple deep learning models. Fig 1 summarizes the differences between CBAM [9], SimAM [11], and PSAFM.

The main contributions are as follows. First, we propose a compact PSAFM module that combines parameter-free pointwise pooling branches with deterministic weighted fusion. Second, we provide a clearer formulation of the channel-statistical attention calculation that converts the fused feature representation into three-dimensional attention weights. Third, we evaluate the proposed PSAFM on CIFAR-10 and CIFAR-100 using ResNet, Pre-ResNet, and MobileNetV2 backbones, and the results show that it improves classification accuracy with limited computational overhead.

## Related work

Backbone architectures have long been improved through changes in depth, connectivity, and computational structure. ResNet introduces residual connections to make deep CNNs easier to optimize [3], while Pre-ResNet further rearranges the residual block to improve gradient propagation [16]. Wide ResNet investigates the effect of widening residual networks as an alternative to simply increasing depth [17]. DenseNet strengthens feature reuse through dense connections [5]. MobileNetV2 uses inverted residual blocks and depthwise separable convolutions for mobile and resource-constrained deployment [18]. EfficientNet scales network depth, width, and resolution in a balanced way [7]. These architectures show that both accuracy and efficiency depend on how feature information is preserved and transformed across the network.

Attention modules provide a complementary route by improving feature selection inside existing backbones. SE is a representative channel attention module that learns channel-wise feature recalibration [8]. CBAM extends this idea by applying channel attention and spatial attention sequentially [9]. Coordinate Attention encodes positional information into channel attention and is especially relevant for mobile networks [10]. The efficient channel attention network (ECA-Net) further simplifies channel attention through local cross-channel interaction [19], and the hybrid attention module (HAM) combines channel attention and spatial attention for image classification [20]. Non-local blocks and Transformer-style self-attention model long-range relationships, although their computational patterns differ from compact CNN attention modules [21,22], and related designs such as GCNet and the dual attention network extend this idea to efficient context modeling and scene segmentation [23,24]. A recent survey summarizes the broad development of visual attention mechanisms and highlights the continuing need for efficient attention designs [12].

Recent studies also show that lightweight feature enhancement remains an active topic. Visual Attention Network explores large-kernel attention for vision backbones [13]. The efficient multi-scale attention module (EMA) introduces cross-spatial learning for multi-scale feature aggregation [14]. SCConv reconstructs spatial and channel features to reduce redundant feature representation [15]. More recently, the efficient local attention (ELA) captures positional information through strip pooling for lightweight networks [25]. These methods confirm the value of spatial-channel modeling, but many of them still use additional convolutional transformations or architectural redesign. In contrast, PSAFM focuses on a simpler plug-and-play setting: it combines parameter-free branch fusion with channel-wise statistical reweighting without adding trainable parameters. In addition to this qualitative discussion, we compare PSAFM with CA, EMA, and ELA experimentally under the same repeated-seed protocol in the Experiments section.

## Method

**Fig 2:**
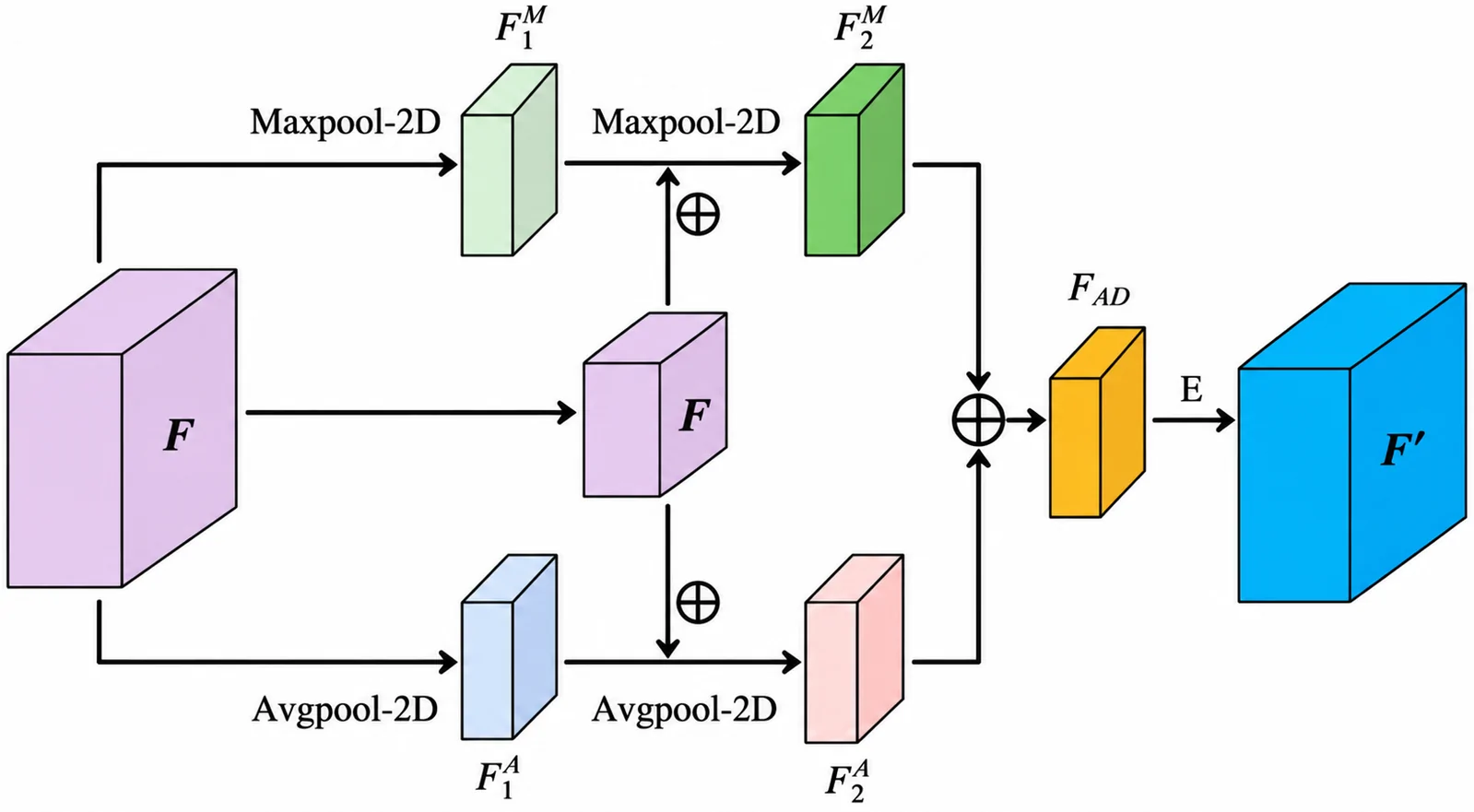
The structure of PSAFM.

This section describes the proposed PSAFM module. We first explain how the pointwise pooling branches in the implementation are fused. We then describe how the fused feature representation is converted into a three-dimensional attention response using channel-wise statistics. Unlike CBAM, which uses additional fully connected and convolutional transformations to compute channel and spatial attention, PSAFM avoids trainable attention branches. Compared with SimAM, PSAFM introduces a deterministic branch-fusion step before statistical attention computation and replaces Sigmoid reweighting with Tanh reweighting. Fig 2 illustrates the overall PSAFM structure.

### Two-dimensional feature extraction

Let $F\in {\mathbb{R}}^{B\times C\times H\times W}$ denote an input feature tensor, where *B*, *C*, *H*, and *W* are the batch size, channel number, height, and width, respectively. Two-dimensional max-pooling and average-pooling are applied in two successive rounds. The first-round outputs are denoted by $F_{1}^{M}$ and $F_{1}^{A}$, and the second-round outputs are denoted by $F_{2}^{M}$ and $F_{2}^{A}$. The operations are written as


$$\begin{array}{c} \begin{array}{ccc} F_{i}^{M} & = & \left\{ \begin{array}{cc} \text{MP-2D}(F), & i=1,\\ \text{MP-2D}(F⊕F_{i-1}^{M}), & i=2, \end{array}\right. \end{array} \end{array}$$ (1)

$$\begin{array}{c} \begin{array}{ccc} F_{i}^{A} & = & \left\{ \begin{array}{cc} \text{AP-2D}(F), & i=1,\\ \text{AP-2D}(F⊕F_{i-1}^{A}), & i=2, \end{array}\right. \end{array} \end{array}$$ (2)

where MP-2D and AP-2D denote two-dimensional max-pooling and average-pooling, respectively, and ⊕ denotes element-wise addition. The two second-round features are fused as


$$\begin{array}{c} F_{AD}=F_{2}^{M}⊕F_{2}^{A}. \end{array}$$ (3)

### Obtaining three-dimensional attention weights

PSAFM then computes an adaptive attention response from channel-wise statistics of the fused feature tensor $F_{AD}$. Let $x_{b,c,h,w}$ denote an element of this fused tensor for batch index *b*, channel index *c*, height index *h*, and width index *w*. The channel mean for each sample and channel is


$$\begin{array}{c} \mu_{b,c}=\frac{1}{H\times W}\sum_{h=1}^{H}\sum_{w=1}^{W}x_{b,c,h,w}. \end{array}$$ (4)

The squared deviation from the channel mean is


$$\begin{array}{c} x_{b,c,h,w}^{square}=(x_{b,c,h,w}-\mu_{b,c}{)}^{2}. \end{array}$$ (5)

The sum of squared deviations over the spatial dimension is


$$\begin{array}{c} S_{b,c}=\sum_{h=1}^{H}\sum_{w=1}^{W}x_{b,c,h,w}^{square}. \end{array}$$ (6)

The normalized response is


$$\begin{array}{c} y_{b,c,h,w}=\frac{x_{b,c,h,w}^{square}}{4\left(\frac{S_{b,c}}{n}+\lambda \right)}+\gamma . \end{array}$$ (7)

where λ is a small regularization constant, γ is set to 0.5, and n=H×W−1. This statistic assigns larger responses to spatial positions whose activation deviates more strongly from the channel mean, while the denominator normalizes the response by the channel-wise spatial variance. Finally, the output is obtained by applying a Tanh activation to the statistic and multiplying it with the fused feature:


$$\begin{array}{c} O_{b,c,h,w}=x_{b,c,h,w}\times \text{Tanh}(y_{b,c,h,w}). \end{array}$$ (8)

Because the attention weight is computed from the current feature statistics, PSAFM can adapt to different inputs without adding trainable parameters. The Tanh activation bounds the reweighting coefficient and helps prevent excessively large attention responses. Since the bounded coefficient is multiplied by the fused feature tensor, the sign information of the original feature activation is preserved.

### Experiments

This section evaluates PSAFM on CIFAR-10 and CIFAR-100 using ResNet [3], Pre-ResNet [16], and MobileNetV2 [18] backbones. We compare PSAFM with the original backbone and three representative attention modules, SE, CBAM, and SimAM, to examine both classification accuracy and computational cost.

## Experimental datasets and setup

### Experimental datasets

The experiments use CIFAR-10 and CIFAR-100 [26]. Both datasets contain 32×32 color images and are standard benchmarks for image classification. CIFAR-10 contains 60,000 images from 10 classes, with 50,000 images for training and 10,000 for testing. CIFAR-100 has the same number of training and test images but contains 100 fine-grained classes grouped into 20 super-classes, making it more challenging for feature extraction and classification. For all models, we follow a common CIFAR training pipeline [27,28]: each image is zero-padded by four pixels on each side, and 32×32 training images are randomly cropped from the padded image or its horizontal flip. Raw images are used during testing. All competing methods were reimplemented in PyTorch [29] to support a fair comparison under a unified implementation.

### Experimental setup

The original main experiments reported in Tables 2 and 3 were conducted on a Lenovo XiaoXinPro 16ACH 2021 laptop and an AutoDL cloud server. The Lenovo XiaoXinPro 16ACH 2021 laptop runs Windows 11 OS and is powered by an R7 5800H processor with 16GB RAM and a 512GB hard drive. On the AutoDL cloud server, the lab environment was configured with Python 3.8.10, PyTorch 1.11.0, using an RTX 2080 Ti as the graphics processing unit (GPU), and CUDA version 11.3. For these main experiments, the training process was carried out for 100 epochs. The base learning rate is set to 0.1 and decayed by a factor of 0.1 in the later training stage. We use stochastic gradient descent (SGD) with momentum 0.9, weight decay 0.0005, 32 data-loading workers, and a batch size of 128. The random seed is set to 1 in the implementation unless otherwise specified.

**Table 2:** Performance comparison of different network architectures and attention modules – CIFAR-10 dataset.

Network	Attention Module	Parameters (Params)	Floating-point operations (FLOPs)	Best accuracy (%)
ResNet-20	Original	0.313114M	0.049859G	91.42
	SE	0.442138M	0.050334G	91.2
	CBAM	0.444718M	0.050529G	91.63
	SimAM	0.313114M	0.049859G	91.4
	PSAFM	0.313115M	0.050031G	92.08
ResNet-56	Original	0.591322M	0.092032G	92.34
	SE	0.849370M	0.092981G	92.66
	CBAM	0.854530M	0.093370G	92.87
	SimAM	0.591322M	0.092032G	92.21
	PSAFM	0.591322M	0.092376G	93.01
ResNet-110	Original	1.147738M	0.176377G	92.51
	SE	1.663834M	0.178274G	93.18
	CBAM	1.674154M	0.179054G	93.25
	SimAM	1.147738M	0.176377G	92.75
	PSAFM	1.147739M	0.177065G	93.3
ResNet-164	Original	1.704154M	0.260721G	92.65
	SE	2.478298M	0.263568G	93.45
	CBAM	2.493778M	0.264737G	93.47
	SimAM	1.704154M	0.260721G	93.24
	PSAFM	1.704154M	0.261754G	92.97
MobileNetV2	Original	2.296922M	0.098055G	89.23
	SE	2.410778M	0.098308G	89.61
	CBAM	2.414358M	0.098726G	89.46
	SimAM	2.296922M	0.098055G	89.16
	PSAFM	2.296923M	0.098330G	91.04
Pre-ResNet-20	Original	0.313594M	0.049859G	90.87
	SE	0.442618M	0.050334G	91.93
	CBAM	0.445198M	0.050529G	91.81
	SimAM	0.313594M	0.049859G	91.41
	PSAFM	0.313595M	0.050031G	92.1
Pre-ResNet-56	Original	0.590426M	0.091573G	91.81
	SE	0.848474M	0.091573G	91.97
	CBAM	0.853634M	0.092912G	92.17
	SimAM	0.590426M	0.091573G	91.95
	PSAFM	0.590426M	0.091745G	93.26
Pre-ResNet-110	Original	1.146842M	0.175918G	93.03
	SE	1.662938M	0.177816G	92.55
	CBAM	1.673258M	0.178595G	92.63
	SimAM	1.146842M	0.175918G	92.77
	PSAFM	1.146842M	0.176606G	93.38
Pre-ResNet-164	Original	1.703258M	0.260263G	93.24
	SE	2.477402M	0.263109G	92.87
	CBAM	2.492882M	0.264279G	93.12
	SimAM	1.703258M	0.260263G	93.17
	PSAFM	1.703259M	0.261295G	93.61

**Table 3:** Performance comparison of different network architectures and attention modules – CIFAR-100 dataset.

Network	Attention Module	Parameters (Params)	Floating-point operations (FLOPs)	Best accuracy (%)
ResNet-20	Original	0.278324M	0.041626G	66.67
	SE	0.286388M	0.041720G	67.13
	CBAM	0.287708M	0.042053G	67.34
	SimAM	0.278324M	0.041626G	66.93
	PSAFM	0.278324M	0.041798G	67.43
ResNet-56	Original	0.861620M	0.127937G	70.45
	SE	0.885812M	0.12822G	71.14
	CBAM	0.889772M	0.129219G	71.15
	SimAM	0.861620M	0.127937G	70.88
	PSAFM	0.861620M	0.128453G	71.37
ResNet-110	Original	1.736564M	0.257403G	70.83
	SE	1.784948M	0.257970G	72.54
	CBAM	1.792868M	0.259968G	72.13
	SimAM	1.736564M	0.257403G	71.44
	PSAFM	1.736564M	0.258435G	71.85
ResNet-164	Original	2.611508M	0.386870G	70.9
	SE	2.684084M	0.387719G	72.54
	CBAM	2.695954M	0.390716G	72.53
	SimAM	2.611508M	0.386870G	71.02
	PSAFM	2.611508M	0.388418G	71.97
MobileNetV2	Original	2.412213M	0.098170G	68.09
	SE	2.526068M	0.098423G	69.52
	CBAM	2.529648M	0.098841G	69.33
	SimAM	2.412212M	0.098170G	67.71
	PSAFM	2.412212M	0.098445G	69.77
Pre-ResNet-20	Original	0.278420M	0.041577G	66.48
	SE	0.286484M	0.041671G	67.13
	CBAM	0.287804M	0.042004G	66.78
	SimAM	0.278420M	0.041577G	66.37
	PSAFM	0.278420M	0.041749G	67.16
Pre-ResNet-56	Original	0.861428M	0.127888G	69.95
	SE	0.885620M	0.128171G	70.66
	CBAM	0.889580M	0.129170G	71.06
	SimAM	0.861428M	0.127888G	69.91
	PSAFM	0.861428M	0.128404G	71.2
Pre-ResNet-110	Original	1.736372M	0.257354G	71.77
	SE	1.784756M	0.257921G	72.08
	CBAM	1.792676M	0.259918G	72.11
	SimAM	1.736372M	0.257354G	71.12
	PSAFM	1.736372M	0.258386G	72.07
Pre-ResNet-164	Original	2.611316M	0.386820G	72.72
	SE	2.683892M	0.387670G	72.73
	CBAM	2.695772M	0.390667G	72.22
	SimAM	2.611316M	0.386820G	72.06
	PSAFM	2.611316M	0.388369G	72.77

### Network configuration

**Fig 3:**
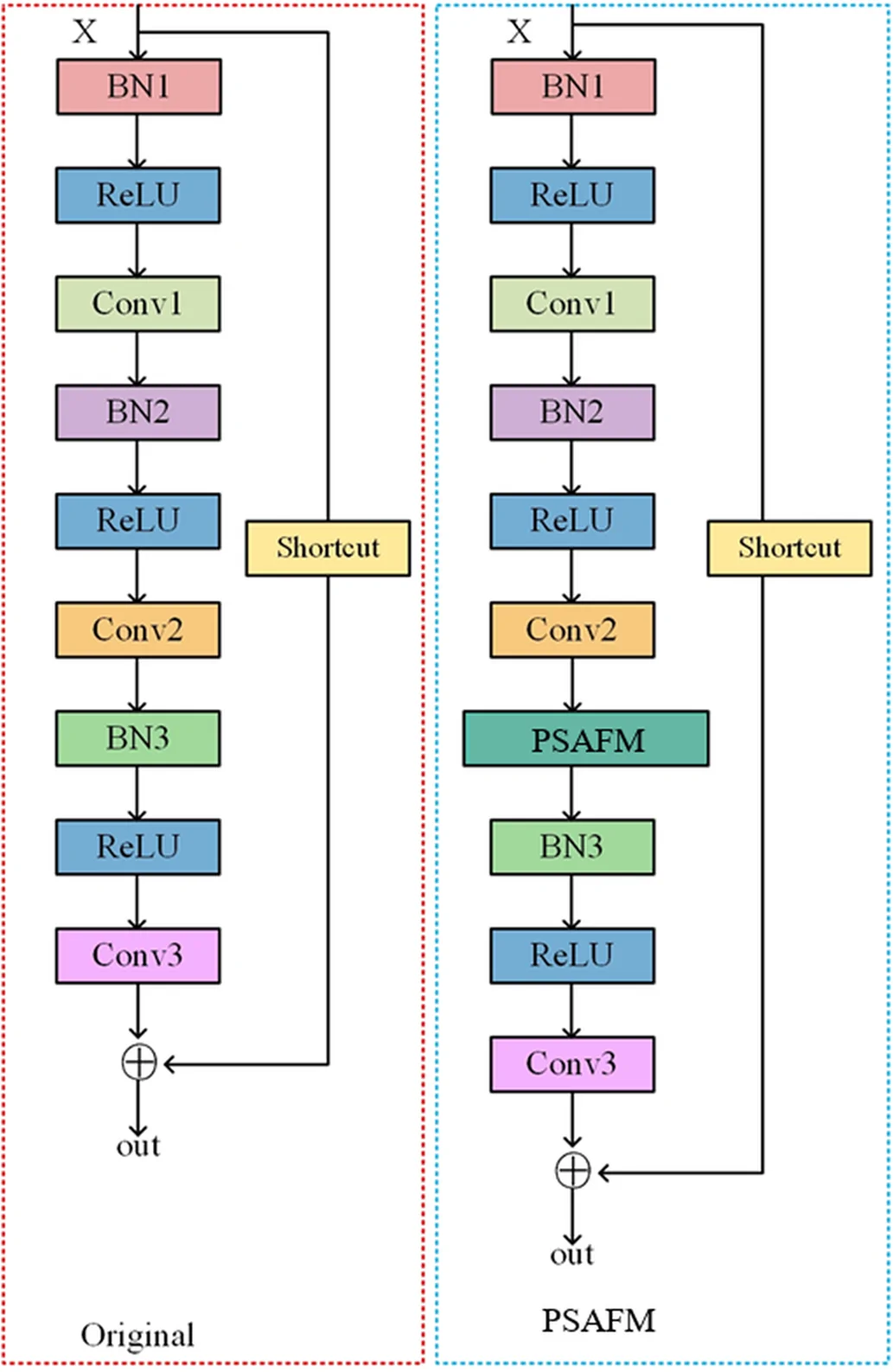
Residual block structures before and after PSAFM insertion. Original denotes the standard Pre-ResNet residual block, and PSAFM denotes the block after inserting the proposed PSAFM module.

To test whether PSAFM can be used as a plug-and-play module, we insert it into the basic or bottleneck residual blocks of ResNet and Pre-ResNet and into the inverted residual blocks of MobileNetV2. The bottleneck residual block of Pre-ResNet is used as an example in Fig 3, which shows the position of PSAFM after the convolutional transformation and before residual feature fusion. This insertion strategy keeps the backbone structure unchanged while allowing PSAFM to recalibrate intermediate features. Because PSAFM does not introduce trainable parameters, any performance gain can be attributed to feature reweighting rather than a larger model capacity.

### CIFAR-10

For CIFAR-10, PSAFM is inserted into the selected ResNet, Pre-ResNet, and MobileNetV2 blocks described above. SE, CBAM, and SimAM are used as comparison modules because they represent channel attention, channel-spatial attention, and parameter-free three-dimensional attention, respectively. The comparison results are shown in Table 2.

Table 2 reports the parameter count, FLOPs, and best accuracy for nine backbone settings on CIFAR-10. PSAFM achieves the best accuracy in 8 of the 9 settings. Compared with the original backbones, PSAFM improves accuracy by an average of 0.85 percentage points. The average improvements over SimAM, SE, and CBAM are 0.74, 0.59, and 0.48 percentage points, respectively. These gains are obtained without an evident increase in trainable parameters: the parameter count is essentially the same as that of the original backbones and SimAM, whereas SE and CBAM introduce additional parameters. The only exception is ResNet-164, for which CBAM and SE reach 93.47% and 93.45%, respectively, while PSAFM reaches 92.97%. Even in this case, PSAFM uses fewer parameters and FLOPs than SE and CBAM. Across all CIFAR-10 settings, PSAFM adds only about 0.37% FLOPs on average over the original backbones while remaining computationally lighter than CBAM and most SE configurations. This result supports the intended design objective: PSAFM improves feature representation while retaining a lightweight, parameter-free structure.

### CIFAR-100

For CIFAR-100, PSAFM is inserted into the same ResNet, Pre-ResNet, and MobileNetV2 blocks as in the CIFAR-10 experiments. SE, CBAM, and SimAM are used as comparison modules. The comparison results are shown in Table 3. Table 3 reports the corresponding CIFAR-100 results. PSAFM achieves the best accuracy in 6 of the 9 settings and improves the original backbones by an average of 0.86 percentage points. The average improvements over SimAM, CBAM, and SE are 0.91, 0.10, and 0.01 percentage points, respectively. These results show that PSAFM remains competitive on the more fine-grained CIFAR-100 task, particularly for ResNet-20, ResNet-56, MobileNetV2, Pre-ResNet-20, Pre-ResNet-56, and Pre-ResNet-164. For some deeper ResNet and Pre-ResNet variants, the advantage of PSAFM is smaller and SE or CBAM achieves slightly higher accuracy. However, these modules also introduce trainable parameters and usually greater computational overhead. In contrast, PSAFM preserves the parameter count of the original backbone and adds only a small amount of computation. The CIFAR-100 results therefore show that PSAFM is attractive when the goal is to improve accuracy while maintaining a compact model profile.

### Ablation study and statistical analysis

**Fig 4:**
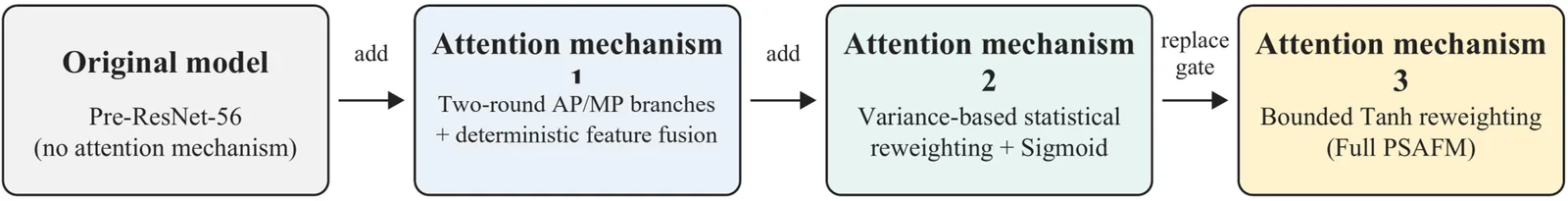
Cumulative workflow of the PSAFM ablation experiment.

**Fig 5:**
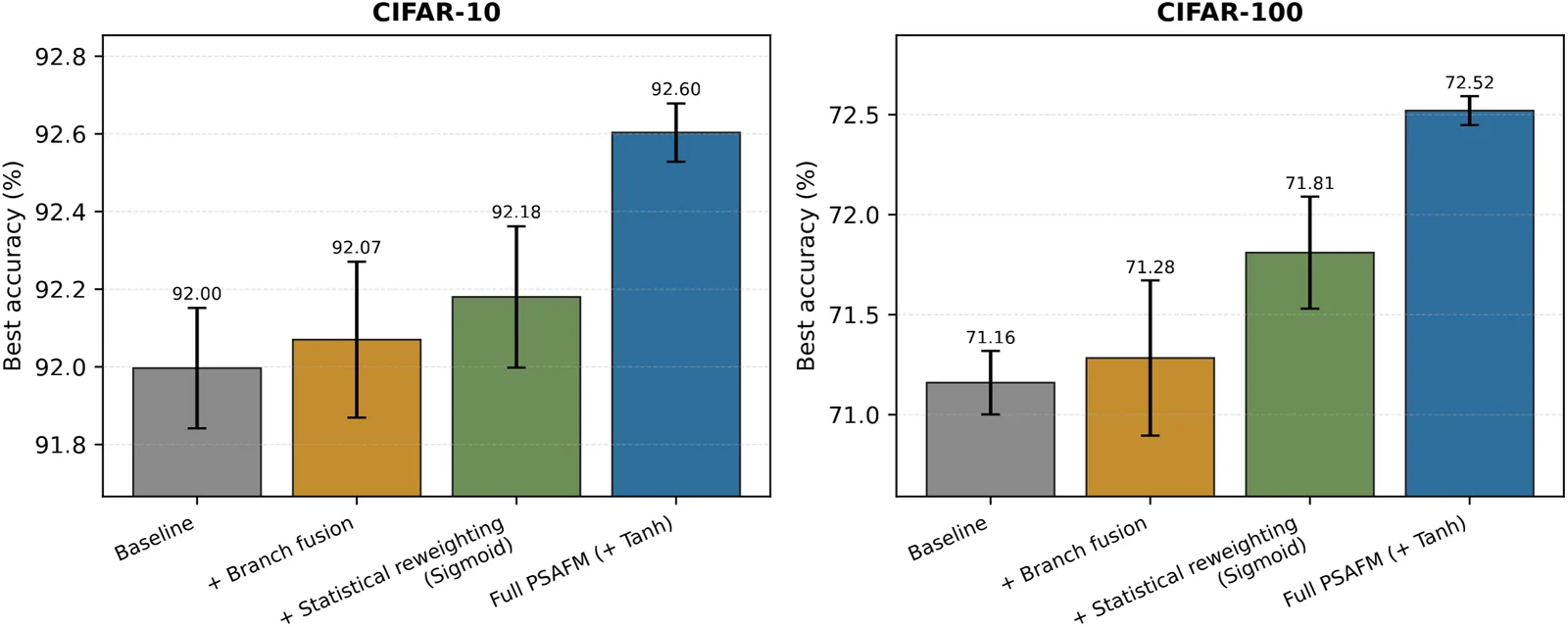
Ablation results on Pre-ResNet-56. Bars show the mean best accuracy across three random seeds, and error bars show the sample standard deviation.

To further examine the contribution of each major PSAFM design step, we conducted ablation experiments using Pre-ResNet-56 on CIFAR-10 and CIFAR-100. The four configurations—the original model (no attention mechanism), attention mechanism 1, attention mechanism 2, and attention mechanism 3 (full PSAFM)—were each trained for 100 epochs with three random seeds using identical data preprocessing, optimizer settings, learning-rate schedule, and batch size; all runs used the same training environment. The original configuration contains no attention mechanism. Attention mechanism 1 adds the two-round average- and max-pooling branches and deterministic feature fusion. Attention mechanism 2 further adds variance-based statistical reweighting with a Sigmoid gate. Attention mechanism 3 replaces the Sigmoid gate with bounded Tanh reweighting and forms the complete PSAFM. Fig 4 presents this cumulative process. Table 4 reports the corresponding results. For each dataset, the table gives the mean best accuracy, mean ± sample standard deviation, and the 95% confidence interval; std denotes the sample standard deviation, and the 95% confidence interval is calculated from the *t* distribution with *t*wo degrees of freedom at the 0.05 level. Fig 5 visualizes the corresponding experimental results.

**Table 4:** Repeated-seed cumulative ablation results for Pre-ResNet-56 on CIFAR-10 and CIFAR-100.

Configuration	CIFAR-10			CIFAR-100		
	Mean	Mean ± std	95% CI	Mean	Mean ± std	95% CI
Original model (no attention mechanism)	92.00	92.00 ± 0.16	[91.61, 92.38]	71.16	71.16 ± 0.16	[70.77, 71.55]
Attention mechanism 1	92.07	92.07 ± 0.20	[91.57, 92.57]	71.28	71.28 ± 0.39	[70.32, 72.25]
Attention mechanism 2	92.18	92.18 ± 0.18	[91.73, 92.63]	71.81	71.81 ± 0.28	[71.11, 72.51]
Attention mechanism 3 (Full PSAFM)	92.60	92.60 ± 0.08	[92.42, 92.79]	72.52	72.52 ± 0.07	[72.34, 72.70]

Table 4 and Fig 5 show that each design step improves the results on both datasets. On CIFAR-10, the accuracy increases from the 92.00% ± 0.16% baseline to 92.07% ± 0.20% after pointwise branch fusion, reaches 92.18% ± 0.18% after adding Sigmoid-gated statistical reweighting, and reaches 92.60% ± 0.08% for the complete module with bounded Tanh reweighting. The first improvement is relatively small compared with the cross-seed variation, whereas the two reweighting steps contribute the largest gains. On CIFAR-100, the corresponding progression is 71.16% ± 0.16%, 71.28% ± 0.39%, 71.81% ± 0.28%, and 72.52% ± 0.07%. Two single-component controls trained under the same protocol support the same conclusion: removing the refined branch from the complete module reduces the mean accuracy to 92.14% ± 0.19% on CIFAR-10 and 72.17% ± 0.41% on CIFAR-100, while using Tanh-based statistical reweighting without branch fusion gives 92.25% ± 0.15% and 72.05% ± 0.12%, respectively. These results show that branch fusion, the refined branch, and variance-normalized Tanh reweighting are complementary. For reference, the 95% confidence interval of the complete PSAFM is [92.42%, 92.79%] on CIFAR-10 and [72.34%, 72.70%] on CIFAR-100.

### Comparison with recent attention mechanisms and statistical analysis

**Fig 6:**
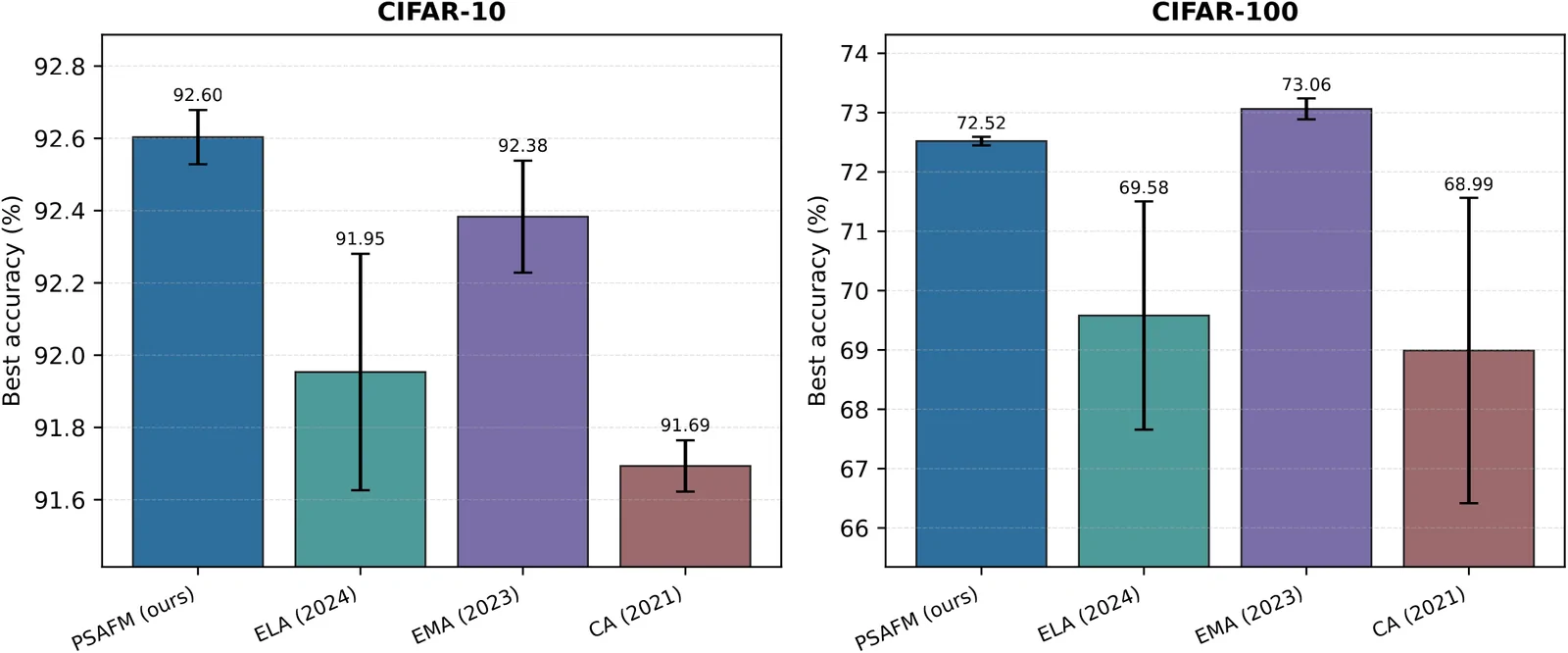
Comparison of PSAFM with recent attention mechanisms (CA, EMA, and ELA) on Pre-ResNet-56 under the repeated-seed protocol. Bars show the mean best accuracy across three random seeds, and error bars show the sample standard deviation.

To further demonstrate the feasibility of the proposed PSAFM, we compared it with the widely used coordinate attention mechanism (CA) [10], efficient multi-scale attention mechanism (EMA) [14], and efficient local attention mechanism (ELA) [25] using the same environment and protocol as the ablation study and statistical analysis. Each module was inserted into the same Pre-ResNet-56 backbone and trained for 100 epochs with three random seeds on CIFAR-10 and CIFAR-100 using identical data preprocessing, optimizer settings, learning-rate schedule, and batch size. Table 5 gives the corresponding experimental results. It reports the mean ± std and 95% confidence interval; std denotes the sample standard deviation, and the 95% confidence interval is calculated from the *t* distribution with *t*wo degrees of freedom at the 0.05 level. Fig 6 visualizes the corresponding results.

**Table 5:** Repeated-seed comparison of lightweight attention modules using Pre-ResNet-56.

Module	Params (M)	FLOPs (G)	CIFAR-10			CIFAR-100		
			Mean	Mean ± std	95% CI	Mean	Mean ± std	95% CI
ELA [25]	0.609242	0.092089	91.95	91.95 ± 0.33	[91.14, 92.77]	69.58	69.58 ± 1.92	[64.80, 74.36]
EMA [14]	0.671738	0.180731	92.38	92.38 ± 0.16	[92.00, 92.77]	73.06	73.06 ± 0.18	[72.63, 73.50]
CA [10]	0.660746	0.094224	91.69	91.69 ± 0.07	[91.52, 91.87]	68.99	68.99 ± 2.57	[62.60, 75.38]
PSAFM (ours)	0.590426	0.091745	92.60	92.60 ± 0.08	[92.42, 92.79]	72.52	72.52 ± 0.07	[72.34, 72.70]

On CIFAR-10, compared with the other modules, the proposed PSAFM achieves the highest mean accuracy (92.60% ± 0.08%) without adding trainable parameters and has the lowest computational overhead. On CIFAR-100, EMA obtains a higher mean accuracy (73.06% versus 72.52% for PSAFM), but it introduces additional trainable parameters and its FLOPs are approximately twice those of PSAFM (0.1807G versus 0.0917G). ELA reaches 91.95% ± 0.33% on CIFAR-10 and 69.58% ± 1.92% on CIFAR-100, while the mean accuracy of CA is lower than that of PSAFM on both datasets. These two coordinate-related modules show noticeably larger cross-seed standard deviations on CIFAR-100 (1.92 for ELA, 2.57 for CA, and 0.07 for PSAFM), indicating greater sensitivity to initialization under this training protocol. PSAFM has the smallest cross-seed variance on CIFAR-100, and its variance on CIFAR-10 is also at a comparably low level. Under this protocol, the mean accuracy of CA is lower than that of the no-attention configuration in Table 4 on both datasets, and ELA is also lower than that configuration on CIFAR-100. The *t*-tests on the three-seed groups show that the advantage of PSAFM over CA on CIFAR-10 is statistically significan*t* (p≈0.0001), and the advantage of EMA over PSAFM on CIFAR-100 is also significant (p≈0.02). Because ELA has a larger cross-seed variance, the advantage of PSAFM over ELA does not reach the 0.05 level (p≈0.07 on CIFAR-10 and p≈0.12 on CIFAR-100); the remaining pairwise differences are also not significant. These results show that, under the strict constraints of fewer trainable parameters and the lowest computational overhead, PSAFM still has an advantage over the compared modules.

In summary, the proposed method is reasonable and effective.

## Conclusion

This paper proposes PSAFM, a parameter-free spatial attention fusion module for convolutional neural networks. PSAFM first uses parameter-free pointwise branch fusion and then computes a three-dimensional attention response from channel feature statistics. Unlike many channel-spatial attention modules, PSAFM introduces no fully connected layers, convolutional attention branches, or additional trainable parameters. Experiments on CIFAR-10 and CIFAR-100 show that PSAFM improves a range of ResNet, Pre-ResNet, and MobileNetV2 backbones with only a small FLOPs overhead. On CIFAR-10, PSAFM achieves the best accuracy in 8 of 9 tested settings and improves the original backbones by an average of 0.85 percentage points. On CIFAR-100, PSAFM achieves the best accuracy in 6 of 9 settings and improves the original backbones by an average of 0.86 percentage points. These results show that parameter-free statistical feature reweighting improves feature representation while maintaining a compact model profile. The ablation experiments on Pre-ResNet-56 further show that the proposed design steps provide consistent accuracy gains over the original no-attention configuration across three random seeds. Under the same repeated-seed protocol, comparison with recent attention mechanisms (CA, EMA, and ELA) further shows that the proposed PSAFM achieves the highest mean accuracy on CIFAR-10 and the smallest cross-seed variance on CIFAR-100 while using the fewest parameters. Compact attention modules can also be useful for practical visual inspection problems, such as industrial defect identification, in which reliable feature extraction and efficient deployment are both important [30]. We also plan to further investigate the proposed PSAFM on other datasets and in detection, segmentation, video understanding, and multimodal vision-language tasks.
